# Bioactivites of *Penicillium citrinum* isolated from a medicinal plant *Swertia chirayita*

**DOI:** 10.1007/s00203-021-02498-x

**Published:** 2021-08-02

**Authors:** Hemant Sharma, Arun Kumar Rai, Rajen Chettri, Poonam Singh Nigam

**Affiliations:** 1 Department of Botany, Sikkim University, 6th Mile Tadong, Gangtok, Sikkim India; 2 Department of Botany, Sikkim Government Science College, Chakung, Sikkim India; 3 Biomedical Sciences Research Institute, Ulster University, Coleraine, Northern Ireland

**Keywords:** Fungal endophytes, Fusarium, Protease, Chitinase, Amylase, Enzymes, Phytopathogen

## Abstract

Endophytes associated with plants have the property to produce active biomolecules with their possible applications in agro-industrial sectors. This study provides a project work on analyzing various activities of fungal endophytes isolated from *Swertia chirayita* of Sikkim Himalayan region. Among several fungal endophytes screened, isolate UTCRF6 was found most active with the secretion of enzymes protease, cellulase, amylase and chitinase, as well as other metabolites Indoleacetic acid and siderophores. This endophyte was found active in restricting the growth of phyto-pathogens, including strains of *Fusarium solani*, *Colletotrichum gloeosporioides*, *Alternaria alternata, Pestalotiopsis theae* and *Sclerotinia sclerotiorum*. Morphological and molecular studies of this endophytic fungus showed similarity with *Penicillium citrinum.*

**Graphic abstract:**

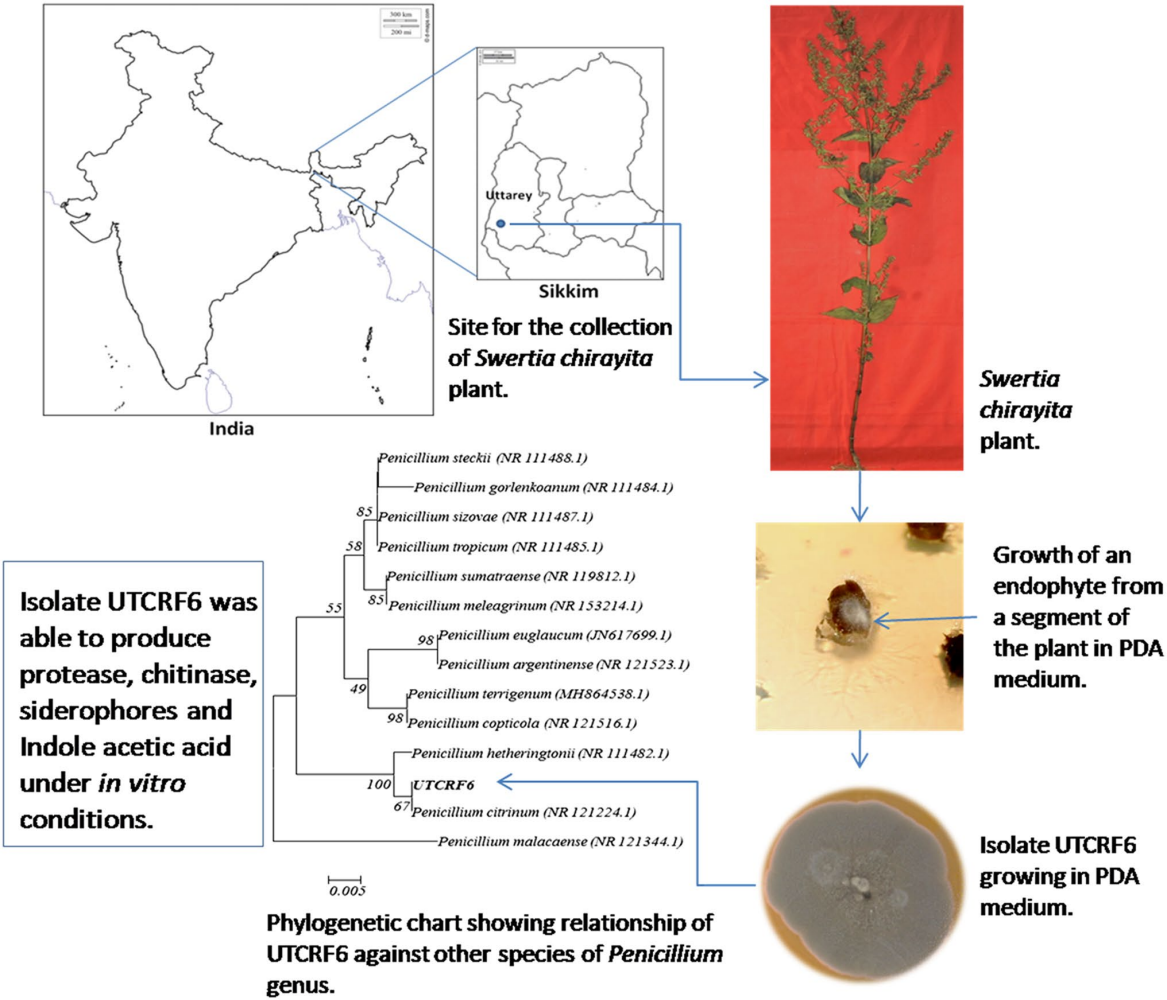

## Introduction

Microorganisms residing within plant tissues without causing apparent harm to their host for the most part of the life cycle are known as endophytes. Endophytic microbes exist in close association with plant tissues maintaining a strong symbiotic relationship. Endophytes have been isolated from every type of plant tissues studied so far (Deshmukh et al. 2020). Symbiotic relationship between plants and fungi date back to the time when terrestrial plants started to colonize the land and fungi may have played a crucial role to facilitate the colonization (Redecker et al. 2000). Apart from plant growth promotion, endophytes have been reported to neutralize invading phytopathogens or produce factors that induce host resistance (Vega 2018). Endo-lichenic fungi represent a relatively untapped bioresource future biopharmaceuticals (Agrawal et al. 2020). These mysterious microorganisms have been associated with the bio-fertilization potential such as solubilization of inorganic phosphates, production of siderophores, and secretion of plant growth-promoting hormones (Zhang et al. 2018). These endophytes also produce important enzymes, such as chitinase, which are useful in solid seafood waste management (Thomas et al. 2020), and also help in inducing host resistance to phytopathogens (Rajulu et al. 2011), as well as produce antifungal metabolites against common phytopathogens (Shentu et al. 2014). These properties of endophytes make them a suitable candidate for accessing their potential in sustainable agriculture and industrial sectors, protecting and utilizing natural bioresources.

S*wertia chirayita* (Roxb. ex Fleming) H. Karst. is a member of Gentianaceae family that typically grows at an elevation of 1200–2100 m above mean sea level and is dispersed across the Himalayan belt from Kashmir to Bhutan and Khasi hills (Kumar and Staden 2016). Among 135 recorded species of *Swertia* genus*,* 40 species are found in India with 8 of them found in Sikkim and surrounding Himalayan region (Envis centre Sikkim, Forest, Environment & Wildlife Management Department, Government of Sikkim, 2011). *Swertia chirayita* is considered significantly superior to other species of the same genus due to its medicinal uses (Kumar and Staden 2016), which may be attributed to the presence of a higher concentration of bioactive metabolites (Kshirsagar et al. 2020). Narrow belt of a suitable geographical habitat and common use in herbal medicines have typically resulted in overexploitation of this species (Pradhan and Badola 2012; Rai et al. 2000). Therefore, an investigation of the endophytes associated with *Swertia chirayita* requires work on their isolation and characterization before the loss of this valuable bioresource from its natural habitat.

Therefore, this project was planned in collaboration with three research institutes (in India and Northern Ireland) and undertaken to secure such valuable microflora and test for their hidden potential. The main aim was to isolate endophytes of *S. chirayita* sampled from the surrounding area of Uttarey located at 35 km from Gyalshing in West district of Sikkim (E 088°08.273’, N 27°28.164’, Elevation 2456 m), and to study screened isolates for their bioactivities with potential applications in sustainable agriculture-resources.

## Materials and methods

### Culture media

Potato Dextrose Agar (HiMedia, India) and Water Agar (Agar, 20.0 g; Distilled water, 1000.0 ml) were used for isolation, sub-culturing and storing of endophytes from its host S*wertia chirayita* samples. Water Agar (WA) and Potato Dextrose Agar (PDA) were supplemented with Streptomycin (50 μg/ml) targeting the growth of fungi and inhibiting bacterial infection. Other media used for screening the endophytes for different activities were: Minimal salt agar (Dipotassium hydrogen phosphate, 7.0 g; Monopotassium phosphate, 2.0 g; Sodium citrate, 0.5 g; Magnesium sulphate, 0.1 g; Ammonium sulphate, 1.0 g; Agar, 15.0 g; Distilled water, 1000.0 ml; pH, 7.0 ± 0.2); Chitinase medium, Pikovskaya’s Agar medium, Starch Agar, Skim Milk Agar, CMCase Agar, Potato Dextrose Broth (all obtained from HiMedia, India).

### Test pathogens

Phytopathogens with relevance to the crops of Sikkim state were procured from the Division of Plant Pathology, Indian Agricultural Research Institute, New Delhi, India. *Fusarium solani* ITCC 7453, *Colletotrichum gloeosporioides* ITCC 5514, *Alternaria alternate* ITCC 7415, *Pestalotiopsis theae* ITCC 6599 and *Sclerotinia sclerotiorum* ITCC 7853 were selected to assess the antagonistic potential of endophytes isolated from *S. chirayita*. All phytopathogens were maintained on PDA slants.

### Sampling and identification of* Swertia chirayita*

With the aim of isolating endophytes healthy growing plants of *Swertia chirayita* (Roxb. ex Fleming) H. Karst. were collected from its natural habitat region across the area of Uttarey West Sikkim, in sterile sampling bags, brought back to the laboratory and stored at 4 ºC until processed. Samples were prepared following the method described by Tucker and Calabrese (2005) and validated with the help of a Botanical Survey of India, Ministry of Environment, Forest and Climate Change, Sikkim Himalayan Regional Centre, and deposited under accession number 0295.

### Processing of plant samples for isolation of endophytes

Processing of *S. chirayita* samples for isolation of endophytes was carried out within 24 h of their sampling. The samples were segregated into leaves, stem and roots. A soft brush was used to remove soil adhering to the roots followed by rinsing all the plant segments with distilled water. Then the surface sterilization process was carried out by a modified method of Anjum and Chandra (2015) under aseptic conditions by soaking in 70% ethanol for one minute, in 5% Sodium Hypochlorite for 10 min and finally rinsing with sterile distilled water few times. The surface sterilization protocol was validated by plating 1 ml of water from the last rinse of the surface-sterilized samples in a suitable medium and observed for any kind of microbial growth during incubation (Pleban et al. 1995).

The outer layer of the surface-sterilized stems and roots was removed, remaining tissues were cut into smaller segments of 50×50 mm size and transferred to nutrient medium PDA. The plates, including those containing water from the last rinse, were then incubated at 28 ± 2 °C. The culture plates were observed regularly for microbial growth arising from the plant sections and any distinctive colonies were sub-cultured in respective nutrient media to obtain axenic fungal cultures, which were then transferred to PDA slopes and stored at 4 °C.

### Isolation and colonization frequency of endophytes

Isolation and colonization frequency of endophytes in the sampled segments of *S. chirayita* were calculated by the method described by Huang et al. (2008). Isolation frequency = Number of microbial isolates obtained from the plant segments/total number of segments inoculated.

Colonization frequency (%) = (Total number of plant segments colonized by endophytic fungi/total number of segments inoculated) × 100.

### Screening the isolated microorganisms for potential activities

#### Phosphate solubilization potential

Loopful of mycelium along with the spores of all isolated endophytes were transferred to Pikovskaya’s Agar medium (PKA) and incubated at 28 ± 2 °C for 5 days. The plates were observed for a zone of clearance around the microbial growth during incubation. Plates showing zone of clearance around fungal colony were considered positive for phosphate solubilization by the respective isolates (Pandey et al. 2006), which were measured for isolates’ phosphate solubilization potential.

#### CMCase activity

Loopful of mycelium along with the spores of all isolated endophytes were inoculated on CMC agar medium [Minimal salt medium supplemented with 0.2% carboxymethyl cellulose (CMC), 1.5% Agar] and incubated at 28 ± 2 °C for 5 days. The microorganisms capable of producing CMCase enzyme were expected to break down carboxymethyl cellulose to simple sugars, a halo around the microbial colonies, indicated CMCase activity (Chang and Yang 2009).

#### Amylase activity

Loopful of mycelium along with the spores of all isolated endophytes were inoculated in a starch agar medium and incubated at 28 ± 2 °C for 5 days. Microorganisms with potential amylase activity were expected to hydrolyze starch into simple sugars, which was detected flooding plates with iodine solution. A halo around the colonies indicated a positive test for amylase enzyme activity in endophyte isolate (Aneja 2003).

#### Siderophores activity

Siderophores activity capability of endophytes was detected using Universal Chrome Azurol Sulphonate (CAS) assay. CAS solution: (a). 10 ml of iron (III) solution (1 mM of FeCl_3_.6H_2_O in 10 mM HCl) was mixed with CAS (60.5 mg in 50 ml of demineralized water); (b). CTAB solution was prepared dissolving 72.9 mg cetrimonium bromide in 40 ml of water. Both a. and b. solutions were mixed slowly to produce a dark blue solution.

PIPES medium: 30.24 g of 1.4- piperazine diethane sulfonic acid (PIPES HiMedia) was dissolved in 900 ml of water, 6 g of NaOH was added to raise the pH to the pKa of PIPES (pH 6.8). 15 g of agar was added before sterilization of the medium.

CAS solution and PIPES media were mixed and poured onto sterile Petri dishes. Loopful of mycelium along with the spores of isolated endophytes were inoculated in the blue-coloured medium and incubated at 28 ± 2 °C for 5 days. The results were interpreted based on the colour change of the medium from blue to orange halo around the inoculated culture due to the iron chelating ability of the microbial isolates (Nagpure et al. 2014).

#### Chitinase activity

Chitinolytic activity of the endophytes was assayed using a modified colloidal chitin medium (Rojas-Avelizapa et al. 1999). Loopful of mycelium along with the spores of the endophytes were inoculated on minimal salt agar plates supplemented with 5% (w/v) chitin. The plates were incubated at 28 ± 2 °C for 5 days followed by staining with 0.1% (w/v) Congo red solution and de-staining with 1 N NaCl solution. Formation of a halo around the microbial colonies indicated a positive result for chitinolytic activity (Nagpure et al. 2014).

#### Protease activity

The protease activity of the microorganisms was screened using Skim milk agar medium. The plates were inoculated with loopful of mycelium along with the spores of endophytes and incubated at 28 ± 2 °C for 5 days. The plates were observed for a zone of clearance around the colonies indicating protease activity in isolates (Aneja 2003).

#### Indoleacetic acid production

Indole Acetic Acid (IAA) production by the endophytes was assayed using a modified protocol of Gordon and Weber (1951). Loopful of mycelium along with spores of the fungal endophytes were inoculated in 50 ml Potato Dextrose Broth (PDB) supplemented with 0.1% L-Tryptophan. After an incubation period of 8 days at 28 ± 2 °C, the culture broth was filtered through sterile Whatman filter paper No. 1 and the filtrate was tested for the presence of IAA using Salkowski reagent. Equal amount of the reagent was mixed with the filtrate and incubated for 30 min in dark. Due to IAA activity pink colour was developed, which was optically measured at 530 nm. Concentration of IAA in the samples was determined from the standard curve of IAA. The mycelium mass retained on filter paper was dried and weighed. IAA production by individual isolates was compared with the dry weight of the mycelium of respective endophytes.

#### Hydrogen cyanide production

Hydrogen cyanide (HCN) has been associated with the induction of systemic resistance in some plants (Wei 1991), and antagonistic nature against phytopathogens (Voisard et al. 1989). HCN production by endophytes was tested using strips of sterile Whatman filter paper soaked in a solution of 0.3% Picric acid and 1.5% sodium carbonate. The paper strips were placed inside PDA slopes inoculated with loopful of mycelium along with the spores of pure culture of endophytes followed by incubation at 28 ± 2 °C after sealing the tubes tightly with parafilm. Results were interpreted based on the colour change of strips from yellow to brown/reddish brown (Potshangbam et al. 2017).

#### Interactions between endophyte and test pathogens

Antagonistic potential of the test endophytes was assayed by Dual culture method. Endophytes were subjected to dual culture assay against phytopathogens: *Fusarium solani* ITCC 7453, *Colletotrichum gloeosporioides* ITCC 5514, *Alternaria alternata* ITCC 7415, *Pestalotiopsis theae* ITCC 6599 and *Sclerotinia sclerotiorum* ITCC 7853 by referring to the types of interactions between an endophyte and a test pathogen described by Chowdhary and Kaushik (2015). The test pathogens and the fungal endophytes were placed at the opposite end on PDA plates and the interaction between cultures was recorded during the incubation period of 7–14 days at 28 ± 2 °C.

### Phylogenetic analysis of the most productive isolate

One of the isolates UTCRF6 found to be the most active and many functionalities was selected for its identification. Its phylogenetic analysis was carried out at National Centre for Microbial Resource (NCMR), National Centre for Cell Science, Pune India. For molecular analysis, genomic DNA was isolated by the standard phenol/chloroform extraction method (Sambrook et al. 1989), followed by PCR amplification of the ITS regions using universal primers ITS1 [5ʹ-TCC GTA GGT GAA CCT GCG G -3ʹ] and ITS4 [5ʹ-TCC TCC GCT TAT TGA TAT GC -3ʹ].

The amplified ITS PCR product was purified by PEG-NaCl precipitation and directly sequenced on an ABI^®^ 3730XL automated DNA sequencer (Applied Biosystems, Inc., Foster City, CA) as per the manufacturer’s instructions. Essentially, sequencing was carried out from both ends so that each position was read at least twice. Lasergene package was used to carry out the assembly followed by NCBI BLAST against sequences from type material for tentative identification (Boratyn et al. 2013). The evolutionary history of the endophyte was inferred using the Neighbor-Joining method (Saitou and Nei 1987). The percentage of replicate trees in which the associated taxa clustered together in the bootstrap test (1000 replicates) were analyzed (Felsenstein 1985). The evolutionary distances were computed using the Jukes-Cantor method (Kimura 1980). The analysis involved 14 nucleotide sequences. All positions with less than 95% site coverage were eliminated and the final dataset was left with a total of 359 positions. MEGA6 was used for evolutionary analyses (Tamura et al. 2013).

### Statistical analysis

All tests were performed in triplicates and descriptive statistics (Mean and standard deviation) were used to interpret the data obtained from enzyme assays and Indole Acetic Acid production. MS Excel was used extensively for all the statistical analysis.

### Results and discussion

*Swertia chirayita* is one of the most used medicinal plant for the preparation of homemade herbal concoctions for various illnesses in the state of Sikkim (Badola and Pradhan 2013). Unaccounted and uncontrolled exploitation of this herb, along with a very narrow range of natural geographical habitat in the Himalayan belt has put a lot of pressure on this precious bioresource. With the loss of this plant species, microbial flora associated with this plant will face the impact of exploitation, which remains unexplored till now. Therefore, our project was planned in collaboration with three research institutes (in India and Northern Ireland) and undertaken to secure such valuable microflora and test for their hidden potential.

We have listed the isolation frequency of endophytic fungi from different segments of *S. chirayita* in Table 1. Maximum number of endophytes were isolated from the root tissue (40%) followed by stems (15.91%) and leaves (7.14%), respectively. Altogether, 21.69% of plant segments were found to be colonized by fungal endophytes. Positive correlation between colonization frequency and isolation frequency was observed.

**Table 1:** Isolation frequency and colonization frequency of endophytic fungi from different segments of *Swertia chirayita*

	Leaves	Stem	Roots	Total
Number of segments inoculated	35	34	34	103
Number of isolates obtained	3	8	12	23
Isolation frequency (numbers)	0.08	0.23	0.35	0.22
Colonization frequency (percent)	8.57	23.52	35.29	22.33

**Fig. 1:**
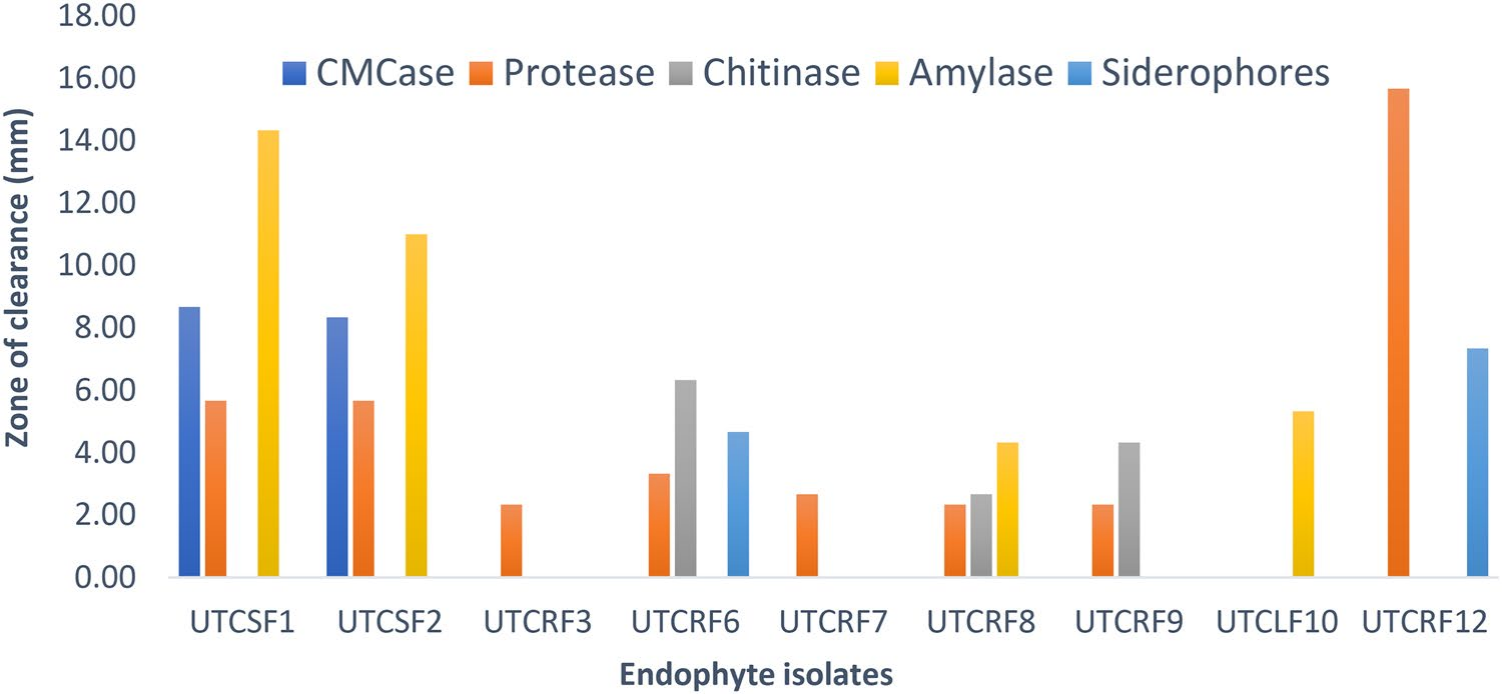
Enzyme and Siderophore activities of endophytes

**Fig. 2:**
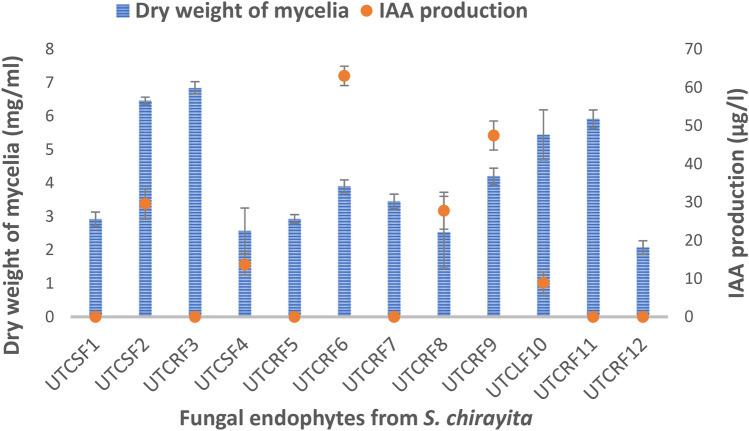
Indoleacetic Acid production by the test endophytes. The amount of IAA produced (µg/ml of growth medium) was compared with the dry biomass of the fungal mat (mg dry weight/ml of nutrient medium). Blue bars represent the dry weight of the fungal mycelia, and red dots represent the amount of IAA produced by the respective endophytes

Total of 36 fungal isolates were obtained from the different segments of *S. chirayita,* of which twelve strains were selected for further study on the basis of distinct morphological characteristics cultured on PDA medium. These were screened for secretion of enzymes of industrial significance CMCase and amylase, protease, chitinase; antimicrobial properties including siderophores and hydrogen cyanide; for the production of plant growth-promoting factors viz*.* IAA and phosphatase. Six of them showed activity for IAA production, eight for protease, three for chitinase enzyme, two for siderophores activity, four for amylase and two isolates were CMCase producers. The results are presented in Figs. 1 and 2, respectively none of the isolates could solubilize inorganic phosphate and produce hydrogen cyanide under in vitro conditions.

Among all endophyte isolates, UTCRF6 was found to be most productive with respect to Indoleacetic Acid synthesis (63.0 ± 2.53 µg/ml) and antimicrobial potential such as protease (3.33 ± 0.58 mm), siderophores (4.67 ± 0.58 mm), and chitinase (6.33 ± 0.58 mm), responsible for inducing host resistance. It is evident from Fig. 1 that few endophytes were better than UTCRF6 in the production of some of the metabolites, however, the isolate UTCRF6 was able to synthesize most of the tested metabolites, with possible significance to their use in agriculture and bioresource management.

IAA produced by different bacterial and fungal endophytes associated with plant growth-promoting ability has been reported earlier (Zhang et al. 2018). Chitinolytic enzymes, produced by endophytes, have been known to trigger host responses against the invading pathogens which may result in increased resistance of the host plant (Zheng et al. 2017). Siderophores activity by endophytes has been associated with their virulence factor, and their synthesis under in vitro conditions has been described by Kajula et al. (2010). Biocontrol potential of protease enzyme is known in fungal strains (Elad and Kapat 1999).

**Fig. 3:**
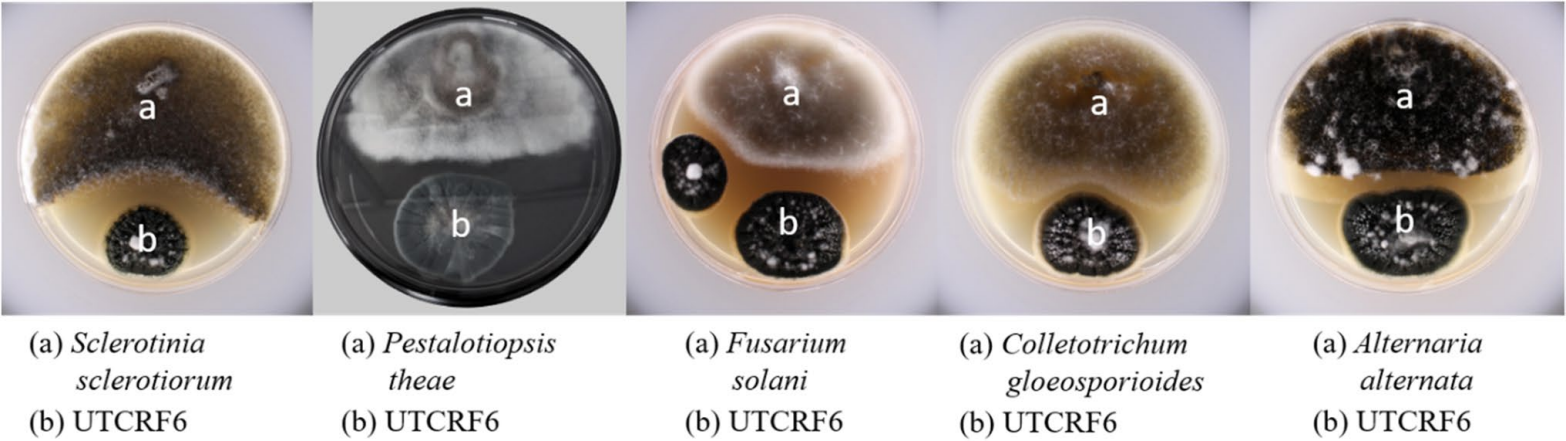
Interaction of endophytes with phytopathogens by dual culture method. Culture conditions: Loopful of inoculum of test pathogen and endophyte isolate was inoculated at the opposite end of a plate containing PDA medium and the results were analyzed after 14 days of incubation at 28 °C

The isolate UTCRF6 was also able to restrict the growth of tested phytopathogens *Fusarium solani* ITCC 7453, *Colletotrichum gloeosporioides* ITCC 5514, *Alternaria alternata* ITCC 7415, *Pestalotiopsis theae* ITCC 6599 and *Sclerotinia sclerotiorum* ITCC 7853 under dual culture assay exhibiting Class 3 interaction, where both pathogen and endophyte grew towards each other and zone of inhibition was formed and maintained for another week (Table 2 and Fig. 3).

**Table 2:** Interactions of *Swertia chitrayita* endophytes with phytopathogenic fungi

Endophyte isolates (1–12)	Type of interaction observed against test phyto-pathogens				
	*Pestalotiopsis theae*	*Colletotrichum gloeosporioides*	*Alternaria alternata*	*Fusarium solani*	*Sclerotinia sclerotiorum*
UTCSF1	Class 3	Class 5	Class 5	Class 3	Class 3
UTCSF2	Class 3	Class 3	Class 5	Class 3	Class 3
UTCRF3	Class 1	Class 5	Class 5	Class 1	Class 1
UTCSF4	Class 5	Class 3	Class 5	Class 5	Class 5
UTCRF5	Class 3	Class 3	Class 5	Class 3	Class 3
UTCRF6	Class 3	Class 3	Class 3	Class 3	Class 3
UTCRF7	Class 7	Class 6	Class 6	Class 6	Class 5
UTCRF8	Class 3	Class 5	Class 5	Class 3	Class 3
UTCRF9	Class 3	Class 3	Class 3	Class 3	Class 3
UTCLF10	Class 3	Class 6	Class 6	Class 6	Class 3
UTCRF11	Class 5	Class 6	Class 6	Class 3	Class 5
UTCRF12	Class 3	Class 7	Class 7	Class 7	Class 7

The test pathogenic organisms used in our dual culture study were selected considering the implications of the pathogens on the food or cash crops of Sikkim. Majority of the farmers residing in Sikkim are highly dependent on cash crops such as large cardamom, buckwheat, mandarin orange, etc. to run their livelihood.

Phytopathogens are known to cause havoc in the agriculture system resulting in heavy financial loses to the farmers. *Colletotrichum gloeosporioides* has been reported to cause severe blight of *Amomum subulatum* (large cardamom) in Sikkim resulting in the total destruction of a high yielding variety of cardamom plant, *Varlangey* (Saju et al. 2013). Similarly, *Alternaria alternata* was responsible for causing leaf spots in Tamarillo (*Cyphomandra betacea*) in Sikkim (Gupta and Choudhary 1994). *A. alternata* was a causative agent for leaf spots disease in Tea as reported from China (Zhou and Xu 2014). *Pestalotiopsis theae* infection of tea leaves is a major concern in Sub Himalayan region of West Bengal (Harikamal et al. 2015). Tea is one of the most popular beverages of the world and infection of the leaves by fungal pathogens such as *A. alternate* and *P. theae* will have a severe impact on the yield. *Sclerotinia sclerotiorum* has been known to infect and cause stem rot in buckwheat plants (Morrall et al. 1976). *Fusarium solani* was isolated from *Citrus reticulata*, one of the major cash crops of Sikkim (Chattopodhyay and Sengupta 1967).

**Fig. 4:**
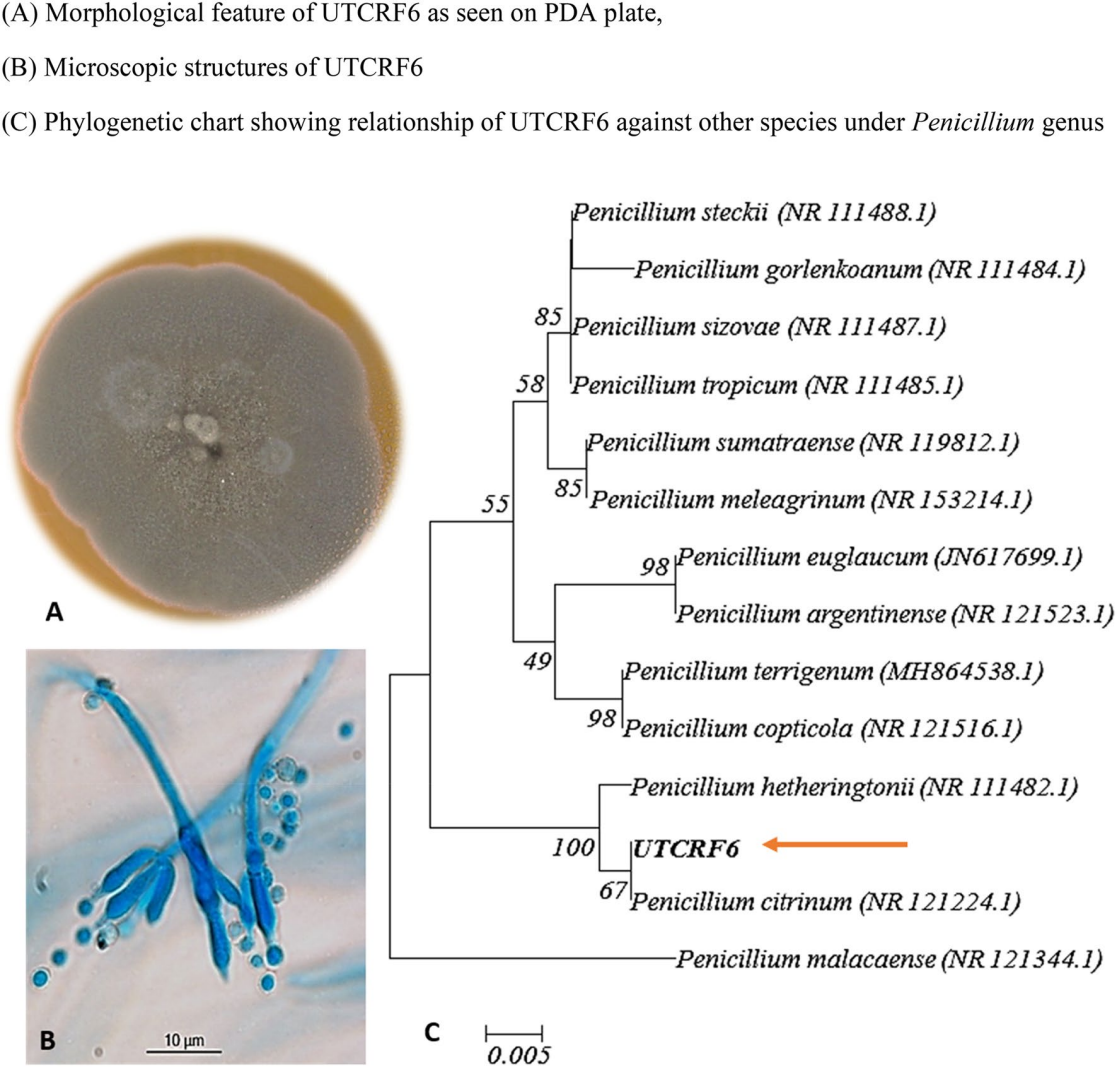
Characterization of endophyte isolate UTCRF6. **A** Morphological feature of UTCRF6 as seen on PDA plate, **B** Microscopic structures of UTCRF6, **C** Phylogenetic chart showing relationship of UTCRF6 against other species under *Penicillium* genus

Microscopic characteristics of the endophyte isolate UTCRF6 are as follows; hyphae: entire, smooth, hyaline with width 6.59 μm, conidiophore: length 13.36 μm and width 2.30 μm, conidia: radius 1.01 μm, phialide: length 10.01 μm and width 2.32 μm, which showed similarity with *Penicillium* sp. Molecular characterization of the endophyte was carried out by sequencing a section of rDNA (using universal primers ITS 1 and ITS 4) and Basic Local Alignment tool (BLAST) of the National Centre for Biotechnology Information was used to match its homology with congeneric species from the database. The sequence of the endophyte had a similarity of 99% to *Penicillium citrinum* which has been submitted to NCBI GenBank under the accession number MK474614. Phylogenetic tree showing the relationship of UTCRF6 with other species of *Penicillium* is shown in Fig. 4. The fungal endophyte (UTCRF6) has been deposited at National Centre for Microbial Resource (NCMR), National Centre for Cell Science, Pune under accession number MCC 1810.

Our report is on the isolation and characterization of *Penicillium citrinum* from a medicinal plant *S. chirayita*, near to its overexploitation*.* In other published reports of *Penicillium citrinum* has been isolated as endophyte from other plants such as *Bruguiera sexangular* and *Scoparia dulcis*, among which one of the strains obtained from *B. sexangular* was found to produce antibacterial metabolite showing antagonistic property against *Bacillus subtilis*, *Micrococcus tetragenus* and *Bacillus cereus* (Zheng et al. 2016). Khan et al. (2008) have isolated a strain of *P. citrinum*, from the roots of *Ixeris repens*, that was able to produce plant growth-promoting hormone, gibberellin, under in vitro conditions. Urooj et al. (2018) have reported several species of *Penicillium,* including *P. citrinum,* as endophytes of various plant species, which were able to suppress root rotting fungi in sunflower when applied alone or along with soil amended with neem cake. *P. citrinum* isolated from *Ceratonia siliqua* was able to produce five novel compounds (El-Neketi et al. 2013). One of the strains of *Penicillium* endophyte*, Penicillium citrinum* LWL4, was able to promote plant growth irrespective of the presence or absence of root rot disease caused by *Sclerotium rolfsii* in sunflower compared to plants under the control group (Waqas et al. 2015). Banana plantlets treated with the strain of endophyte, *P. citrinum* BTF08, showed delayed progression of symptoms, lower rate of disease occurrence and disease severity when infected with *Fusarium oxysporum* f. sp. cubense race 4 (FocR4) (Ting et al. 2012).

### Conclusion

*S. chirayita* were found to harbour many fungal endophytes, their isolation and characterization is reported in this paper first time. Qualitative screening of all endophytes under in vitro conditions exhibited useful characteristics with their possible applications in agriculture. Among all endophyte-isolates, UTCRF6 identified as *Penicillium citrinum* was found to be most prolific with the production of chitinase and protease enzymes, and was able to restrict the growth of several phytopathogens. Biosynthesis of enzymes, antimicrobial property, production of host secondary metabolites, studied in this project, ascertains the potential of endophyte isolates for further study on a larger scale for application of their bioactivities.
